# Multicomponent Reading Intervention: A Practitioner’s Guide

**DOI:** 10.1002/trtr.2265

**Published:** 2023-11-22

**Authors:** Johny Daniel, Amy Barth, Ethan Ankrum

**Affiliations:** Durham University, Durham, UK; William Jewell College, Liberty, MO, USA; Creighton University, Omaha, NE, USA

## Abstract

Discover the effective approach of multicomponent reading interventions, a personalized approach to support elementary grade students with reading difficulties. Explore how teachers can combine word reading, fluency, and comprehension to empower students’ reading journey.

Ms. Patel, the dedicated special education teacher, gathers her Grade 4 students with reading difficulties for their small-group session. She has been diligently implementing phonics instruction, and she observes that the students are making strides in reading monosyllabic words. However, she also notices that their progress in tackling multisyllabic words has hit a road-block. Additionally, they still require support in comprehending the meaning of the text, and their reading fluency remains laborious. Determined to find effective solutions, Ms. Patel recently came across the concept of multicomponent reading interventions. Excited about its potential benefits, she decides to introduce this approach to her small group. Her plan involves targeting multiple reading-related skills simultaneously during each session, with the aim of accelerating her students’ reading progress. With enthusiasm and dedication, Ms. Patel sets out to design and implement the multicomponent intervention.

## What is a Multicomponent Reading Intervention and Why is it Needed?

Students who demonstrate reading levels that are below their age-group peers are generally referred to as students with reading difficulties or struggling readers (e.g., Scammacca et al., 2015). This population encompasses children who have been diagnosed with a learning disability, such as dyslexia, due to their low performance on various reading assessments. Researchers in the field of reading education have endeavored to explore and understand the reading profiles of children with reading difficulties to help guide intervention efforts to meet students’ individual needs (e.g., Leach et al., 2003). One consistent finding across a range of past research studies is that elementary grade students with reading difficulties exhibit diverse areas of reading needs (e.g., Daniel & Barth, 2023; Leach et al., 2003; Miciak et al., 2022). These studies demonstrate that, compared to peers, students with reading difficulties may face challenges in word reading, reading comprehension, reading fluency, vocabulary knowledge, inferencemaking, and/or oral comprehension.

For instance, Miciak et al. (2022) reported that Grade 3 and 4 children with reading difficulties performed significantly below their typical peers in all areas of reading (i.e., word reading, reading fluency, reading comprehension, vocabulary, and knowledge of syntax). Daniel & Barth, (2023) also reported similar findings in a sample of Grade 5 and 6 students, where children with reading difficulties demonstrated below-average scores in reading comprehension, word reading, vocabulary knowledge, and oral comprehension skills.

Given that children with reading difficulties may have needs in all reading domains, over the last decade, researchers have experimented with implementing multicomponent reading interventions that target all areas of reading during supplemental small-group instructional sessions (e.g., Vaughn et al., 2022; Wolf et al., 2009). Data from intervention studies suggests a positive effect of multicomponent reading intervention on students’ reading outcomes (e.g., Denton et al., 2022; Scammacca et al., 2015; Vaughn et al., 2022). Thus, this article aims to outline how teachers and teaching assistants can develop and customize multicomponent reading interventions to meet the needs of a diverse group of learners.

## How to Design and Implement a Multicomponent Reading Intervention

This section describes instruction for a small group of Grade 4 students who perform below grade-level benchmarks on word reading, reading fluency, and reading comprehension assessments. The example demonstrates how various reading-related components can be embedded in a single lesson that can be taught outside the mainstream classroom in small-group settings (i.e., two to five students). For each component, theories and research that support their use are described, followed by explanations of how the lesson can be implemented with students. Additionally, examples are provided on how teachers could potentially provide corrective feedback during lessons and monitor students’ progress.

### Word Reading + Word Meaning Instruction

#### Theory and Research.

**Figure 1:**
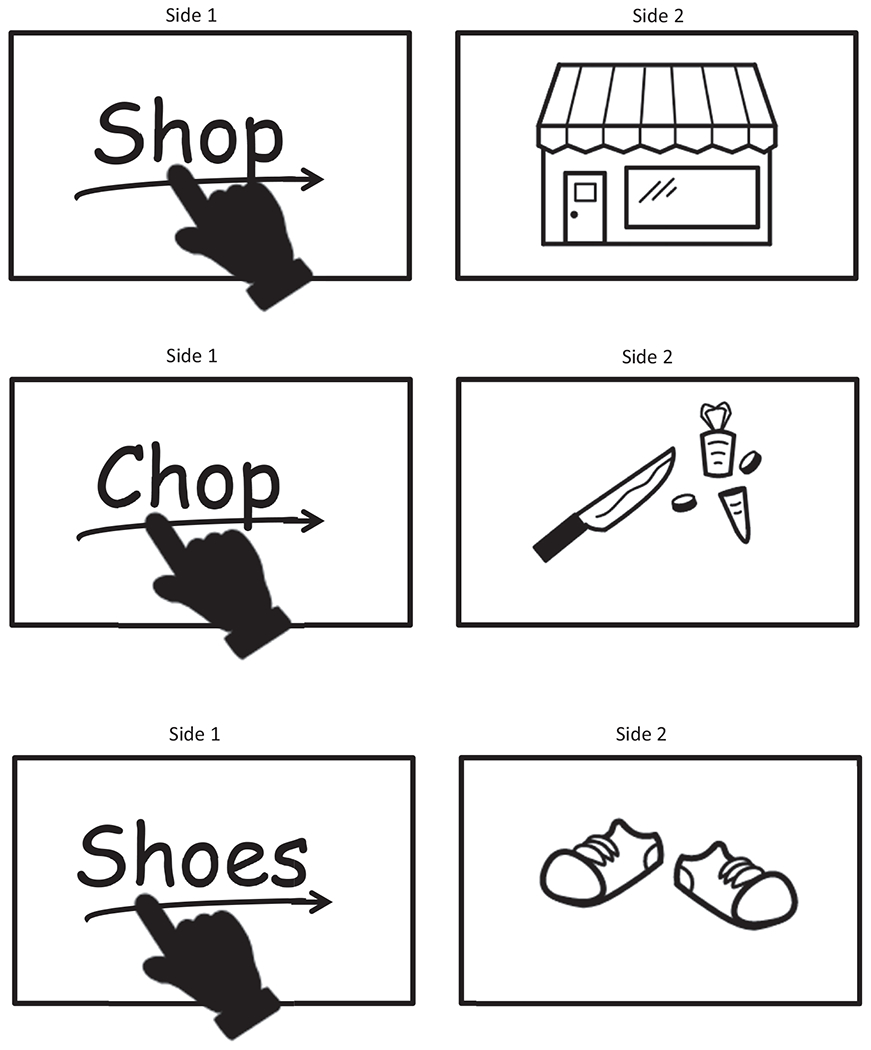
Word Card Examples

The practice of teaching word reading along with word meaning has theoretical underpinnings. The lexical quality hypothesis (Perfetti, 2007; Perfetti & Hart, 2002) suggests that knowledge of word forms and word meanings allows individuals to identify words and reliably connect them to their correct contextual meaning, which is key to reading comprehension. Researchers examining reading development have demonstrated that students’ semantic knowledge of words impacts their word reading ability through a “division of labor” (Steacy & Compton, 2019; Taylor & Perfetti, 2016). The division of labor allows the reader to recognize unknown words through accessing the phonological, orthographical, and/or semantic knowledge of the word. In a recent intervention study, researchers demonstrated that teaching word meaning along with word reading had a greater impact on treatment group students’ word reading fluency compared to the comparison group that was only taught word reading skills (Austin et al., 2021). In addition, there is some evidence to supports the use of images to increase mastery of words among elementary school children with word reading difficulties (Steacy & Compton, 2019). See Figure 1 for an example word reading lesson with image cards.

Additionally, as student progress from elementary to middle school, there is a significant change in the complexity of words they read (Hiebert et al., 2005). It has been reported that students with reading difficulties who learn to decode monosyllabic words fluently, can often demonstrate challenges in reading multisyllabic words (Duncan & Seymour, 2003; Toste et al., 2016). This could be because multisyllabic words present challenges for reading due to their length, as well as factors like syllable division, word stress, uncertain vowel pronunciations, complex grapheme-phoneme correspondences, and intricate word structures (Heggie & Wade-Woolley, 2017). Below is an example of a multisyllabic word reading and word meaning lesson that can be one part of a multicomponent lesson.

#### Goal Setting.

The teacher sets the purpose for the activity, stating “*Today we’ll explore how words are sometimes made of meaningful parts called morphemes. Understanding morphemes helps us figure out meaning of words we don’t know yet. Let’s get started*.”

#### Explicit Instruction.

First, the teacher writes the prefix *un* on an interactive whiteboard, says “*the letters u-n, make the sound /un/, what sound*? She has each student repeat /un/. Next, the teacher shows the written word ‘unable’ on the interactive whiteboard, states clearly that the prefix *un* makes the sound /*u*//n/ and uses her finger to follow and sound out each phoneme in the word, /*u*/ /*n*/ /*a*//*b*//*l*/. The teacher then blends the sounds to say the word out loud *unable*.”

**Figure 2:**
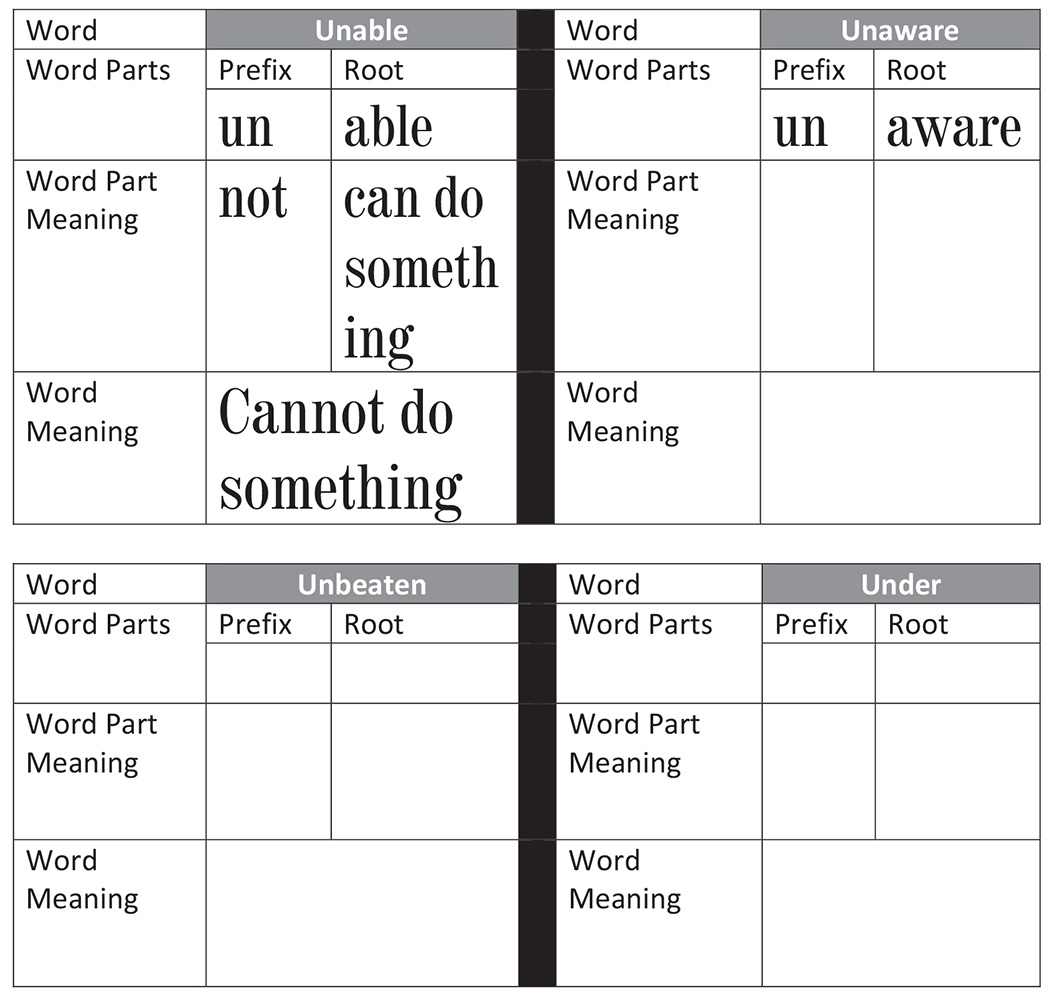
Morpheme Analysis: Study the Word Parts to Figure out Meaning of Words *Note*. From Reading Resource Centre Lesson 41 to 50 (https://www.readingresourcecentre.org/multicomponent-lessons).

As shown in Figure 2, the teacher then introduces a morphological analysis worksheet and models the activity. She first introduces, the meaning of the prefix *un* stating that *the morpheme un means* “not.” “*Let’s write this information on the worksheet*.” Once students write “not” in the word part meaning of the prefix section, the teacher then introduces the concept of root word. She says, “*the remaining part of the word, without the prefix is the root word. Here the root word is able. Able means to have the skills or power to do something. Let’s write this information in the worksheet*.” After students have written the meaning of the root word, the teacher then shows students how adding the prefix changes the meaning of the word. She says, “*Adding the prefix ‘un’ to the root word ‘able’ changes the meaning of the root word. Remember un means not, so unable means not able to do something*.”

The teacher then provides guided practice for the next two words unaware and unbeaten. She also introduces the word *under* as a nonexample. She says, “*when I cover the word un, der is left. Der is not a real or meaningful root word. If you come across a word that does not have a meaningful root word, then in that words the letters un are not morphemes and do no mean not*.” Finally, the teacher provides students with other example words starting with the morpheme *un* for independent practice. Note, if students are working in small groups, it may be beneficial to pair students during guided or independent practice (see Fuchs & Fuchs, 2005; Fulk & King, 2001).

#### Corrective Feedback.

If a student makes an error in pronouncing a word, here is how teachers can provide corrective feedback. “*That’s not quite right. Let’s review this word again. Listen, /u/ /n/ /f/ /i/ /t/ is unfit. What’s the word?*”

#### Progress Monitoring.

Teachers can assess multisyllabic word reading and word meaning growth by providing students with a list of 10 words that start with the same morpheme and have them read those words out loud to assess accuracy (see Appendix A) (Diamond & Thorsnes, 2018). If a student is unable to pronounce a word, teachers should encourage students to pronounce the word but after 5-seconds of waiting they should read the word out loud. Additionally, teachers can pair students, with one student recording the accuracy while the other reads the word list.

Word meaning on the other hand can be assessed using multiple methods. One way would be to present students with a list of words and their meanings and have students match the word to their meaning. Another way would be to have students identify the closest synonym of a highlighted word or morpheme from a list of three (e.g., **shut**–open, close, share; **pre**–post, during, before) (Diamond & Thorsnes, 2018).

### Reading Fluency Instruction

#### Theory and Research.

Reading fluency refers to reading connected text with speed and accuracy. Several past studies have identified reading fluency as a key indicator of students’ success in reading comprehension (e.g., Stevens et al., 2017; Therrien, 2004). Theoretical frameworks, such as the verbal efficiency theory (Perfetti, 1992), suggest that when word reading is laborious and error-prone, more cognitive resources are dedicated to reading the text while less cognitive resources are available to comprehend the meaning of the text. There is research that also demonstrates that elementary grade students’ reading fluency skills can be a good indicator of their overall reading competence (Jenkins et al., 2003). Thus, reading fluency is an integral building block of reading development, and increasing students’ fluent and accurate reading can prepare them to read and comprehend more complex grade-level texts.

For reading fluency activities, it is recommended to select a passage that is at the student’s independent reading level (i.e., a text the child can read with at least 95% accuracy; Rasinski & Padak, 2013). For instance, if students in the group are reading at Grade 1 level, then a Grade 1 level text can be selected. Several websites such as Readworks (https://www.readworks.org) and Newsela (https://www.newsela.com) provide grade-level texts that teachers can use. The fluency texts should be approximately 150–250 words in length.

#### Goal Setting.

The teacher tells the students, “*Today we are going to read about (insert topic), and our goal is to practice reading fluently. To read fluently means to read with accuracy, speed, and expression. I’ll read the passage first and have you use your fingers to follow along on your sheet. I will leave out some words that I will have you read out loud*.”

#### Explicit Instruction.

Students should engage with the fluency text at least two to three times (Therrien & Kubina, 2006). The first time, the teacher models by reading the text out loud, and students use their fingers to follow along with their own copy of the text. The teacher may omit certain words, prompting students to read those words aloud. Next, students can read the text out loud individually or in pairs with their small-group peers. Finally, students can engage in whisper reading the text.

It might be helpful to prepare at least two reading passages for this section. If a student completes the task early, a second passage can be used to target reading fluency as well. Furthermore, if teachers are working in small groups, they can implement peer work to have students time their peers and provide corrective feedback (see Fuchs & Fuchs, 2005; Fulk & King, 2001).

#### Corrective Feedback.

If students mispronounce a word, teachers can again break the word into its individual phonemes/syllables and have the student repeat the word. It may also be helpful to have a peer or teacher model fluent reading if a student is reading the text laboriously.

#### Progress Monitoring.

A commonly administered reading-related progress-monitoring measure is the oral reading fluency assessment (Honig et al., 2018). Students are asked to read a passage quickly and accurately for one-minute. Teachers record any errors students make and calculate the words correct per minute (i.e., Total Words Read–Errors = Words Correct Per Minute). Teachers or students can graph the words correct per minute score on a weekly basis to track their growth in reading fluency (Lemons et al., 2014). There is also empirical data suggesting that graphing students’ oral reading fluency scores can help enhance their self-determination and improve their oral reading fluency (see Didion et al., 2020).

### Instruction to Improve Reading Comprehension

#### Theory and Research.

In this section, students read and demonstrate their comprehension of grade-level text. The current recommendation in the field is for students to read texts that present grade-level concepts and ideas to ensure that the knowledge gap between students with reading difficulties and their typical peers can be bridged (Vaughn et al., 2022). The recommendation also is to scaffold these reading activities to ensure student success; each reading can be completed over two sessions to give students an opportunity to re-read the content before answering comprehension questions (Vaughn et al., 2022). This section describes how students can learn to use keywords to write the main idea statements to demonstrate their comprehension of the text (Hagaman et al., 2010; Williams, 1986).

#### Goal Setting.

The teacher sets a purpose for reading. In the example reading, the teacher states, “*Today we are going to read a story about Abraham Lincoln when he was a young man, before he became the president of United States*.”

#### Explicit Instruction.

**Figure 3:**
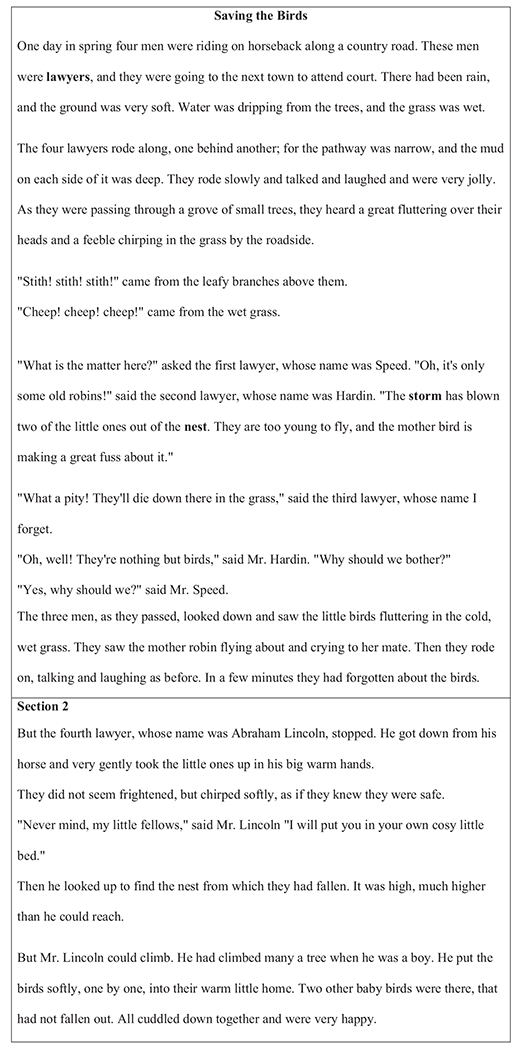

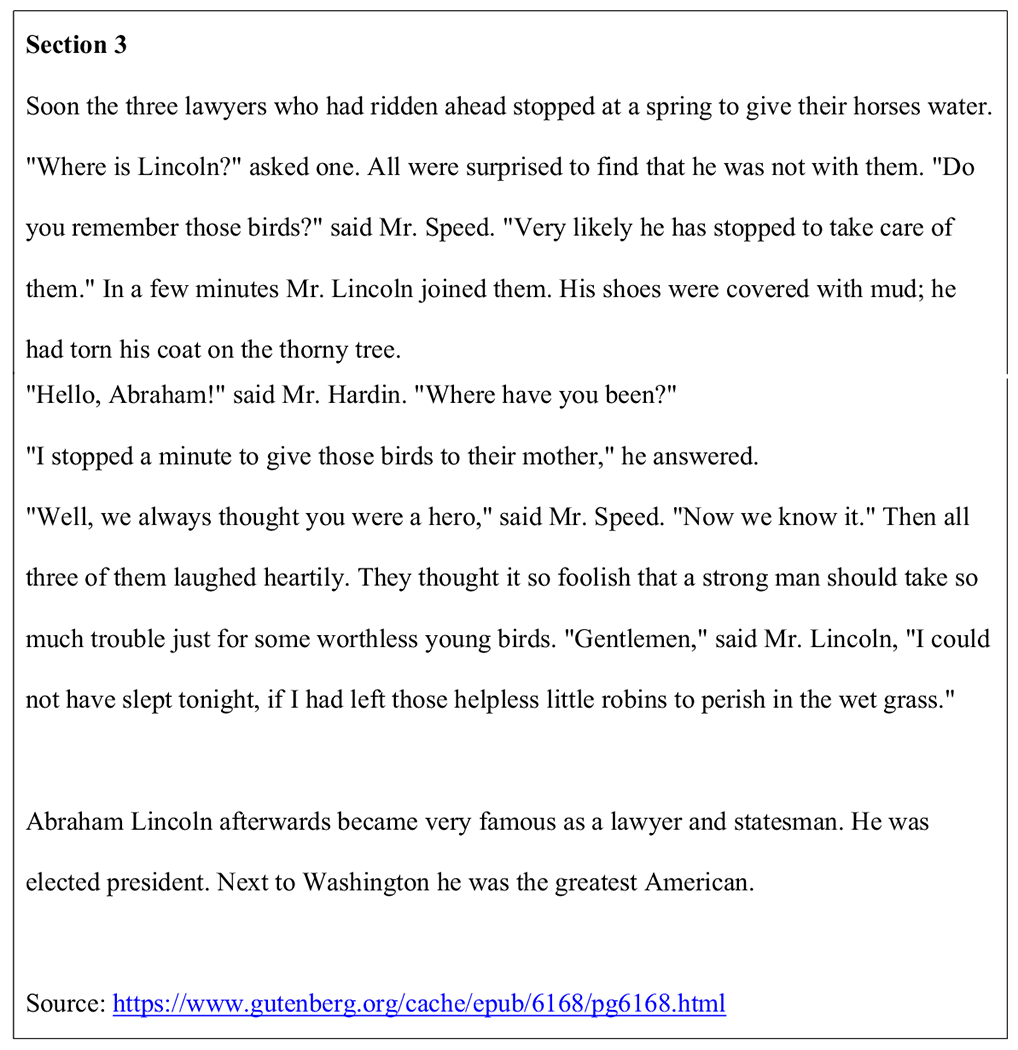
Grade-Level Reading Passage Example

Larger and longer texts should be broken into smaller sections for students to reflect on their understanding of each section before moving on to the next section. Additionally, if there are one or two keywords that are key to comprehending the text, those should be taught explicitly before the reading activity (Beck et al., 2013). In the reading example in Figure 3, the teacher chooses to read section 1 along with her students. Each student reads one sentence as the teacher whips around her small group.

After reading, the teacher asks, “*Who is this passage mostly about?*” The teacher models the response by thinking out loud: “*This passage is mostly about the lawyers*.” Next, the teacher asks, “*What are some keywords that describe the passage*?” The teacher explains that keywords highlight the most important ideas. “*When we look for these important or key words, it makes it easier for us to figure out what the passage is about and write a summary statement*.” The keywords for this passage (highlighted in bold) are lawyers, storm, and nest. Then, the teacher inquires, “What is the most important idea about the lawyers in this passage?” To write a main idea statement, the teacher suggests using the highlighted keywords: “The lawyers noticed that the storm has blown some chicks out of their nests.” The teacher waits for students to write it down and checks to ensure all the keywords are included in the main idea sentence.

For the second section, the teacher chooses choral reading, getting the entire small group to read the section out loud. At the end of the section, the teacher asks her students to identify three keywords and use those keywords in a main idea statement. Here the teacher provides guided practice by asking students to identify the keywords and providing corrective feedback. The teacher then asks students to write a main idea statement using the identified keywords. The teacher asks students to read their main idea statements and provides corrective feedback and/or positive praise for well-written statements. For the third section, the teacher uses paired reading with each student reading one paragraph. The teacher completes this section by asking the keyword and main idea questions and providing corrective feedback when necessary.

The following day, students are expected to read the text silently on their own and answer multiple choice comprehension questions at the end of each section and answer inference questions after reading all sections. Further, students are asked to write a main idea statement for the entire passage. It would be beneficial for teachers to provide corrective feedback when needed. The purpose of multiple readings of the same text is to ensure that students have an opportunity to build background knowledge about the content to help ensure success when answering the comprehension questions (see Vaughn et al., 2022).

#### Corrective Feedback.

When students encounter challenges in writing main idea statements, the teacher approaches the situation with a focus on students’ strengths. The teacher first ensures if students have identified the relevant keywords, supporting them in this process through guided practice. If needed, the teacher models how students can utilize these keywords to construct a concise main idea statement, recognizing the individual needs and abilities of each student. Implementing think-aloud can be a valuable resource to empower students in their learning journey (Davey, 1983).

#### Progress Monitoring.

There are multiple ways for teachers to assess students’ progress in comprehending grade-level text. (1) Teachers may administer a grade-level Maze assessment where students read a passage and for every seventh word they choose from a list of three words to decide which word makes to most sense in the sentence (Guthrie et al., 1974). (2) Teachers may also have students read a short passage and identify the keywords and write a main idea statement (Williams, 1986). (3) In addition, teachers may create a list of sentences that students read and identify if the sentence makes sense or not (see Massey, 2003; Oakhill et al., 2015). For instance, students read the sentence: *Three lawyers were on their way to cart*. They would be expected to state that the sentence does not make sense and share how they would correct the sentence (i.e., replace the word *cart* with *court*).

## Putting It All Together

Table 1 shows the various components, their importance, and the average reported effect size on reading comprehension outcomes across different reviews. Thus, by combining word reading, word meaning, reading fluency, and reading comprehension instruction, teachers can create engaging and personalized lessons to support their students’ reading development. To begin the multicomponent lesson, teachers may consider starting with an activity that introduces either a phonics lesson or a morphemic analysis lesson. This segment could last for approximately 10 min and is essential for building foundational word reading skills that can improve students’ response to multicomponent reading interventions (Daniel et al., 2021; Vaughn et al., 2020). Notably, explicit instruction and examples play a crucial role in helping students grasp phonemes, graphemes, and morphemes, along with their corresponding sounds and meanings (Mesmer & Griffith, 2005). When students practice with guidance first and then independently, it reinforces their understanding (see Archer & Hughes, 2010).

Following the word reading component, the next section, which could take around 5 min, could focus on reading fluency instruction. Teachers could select a passage at the students’ independent reading level and model fluent reading for them. Students can then engage in repeated readings, either individually or in pairs, to improve their reading speed, accuracy, and expression (Therrien & Kubina, 2006).

After addressing word reading and reading fluency, the remaining supplemental instructional time (15–20 min) could be devoted to reading comprehension instruction. In this segment, teachers may choose appropriately challenging texts, scaffold the reading activity by breaking the text into smaller sections, and explicitly teach a reading comprehension strategy such as identifying the main idea. Prior to reading each section, teachers might explicitly teach keywords that are vital for comprehension, and a reading strategy to help structure the reading task (Beck et al., 2013).

During the reading activity, students can participate in various reading methods, such as choral reading, paired reading, and silent reading. For each section, teachers can ask questions about the main idea and the identified keywords, encouraging students to formulate concise main idea statements. Providing corrective feedback and praise for well-written statements can further support their comprehension development (Pany & McCoy, 1988). Throughout the intervention, students might engage with the text multiple times to build background knowledge and enhance their comprehension skills (Vaughn et al., 2022). Teachers can use various progress-monitoring assessments, such as Maze assessments, main idea statements, oral reading fluency, and sentence comprehension activities, to track students’ growth in each component.

It’s important for teachers to remember that the suggested time frames are just rough guides, and flexibility is crucial to meet the specific needs of individual students. Implementing the multicomponent reading intervention in small-group settings (i.e., two to five students) allows teachers to provide targeted and personalized instruction, empowering students in their reading journey. By combining these various reading-related components and providing students with agency, teachers can create engaging, effective, and personalized lessons that cater to the unique needs of upper elementary students with reading difficulties. The multicomponent intervention aims to bridge the knowledge gap between students with reading difficulties and their peers, empowering students to become proficient and confident readers.

## Figures and Tables

**Table 1:** Summary of Various Reading Components

Reading component	Importance	Recommendations	Component’s effect on Reading comprehension outcomes
Word reading + word meaning instruction	■ Essential foundational skill for reading growth	■ Provide regular instruction on decoding familiar and unfamiliar words	Word reading instruction yielded positive improvements in students’ reading comprehension outcomes, with reported effects ranging from 0.05 to 0.12 (Vaughn et al., 2022)
	■ Especially vital for upper elementary and later year students with reading difficulties as instruction shifts from learning to read to reading to learn	■ Connect word reading instruction with word meaning instruction to improve lexicon quality	
	■ Inadequate instruction can impede comprehension of grade-level text	■ Include word reading tasks for decoding multisyllabic words and working with peers to improve multisyllabic word reading skills	

Reading fluency instruction	■ Improving reading fluency can aid the process of reading comprehension	■ Use independent reading level texts to improve confidence and fluency	Reading fluency instruction yielded positive improvements in students’ reading comprehension outcomes, with reported effects ranging from 0.21 to 0.75 (Stevens et al., 2017; Therrien, 2004)
		■ Repeated reading can support growth in reading fluency	
		■ Progress-monitoring graph show students their fluency growth and can keep them motivated	

Reading Comprehension Strategies	■ Using grade-level texts ensures content knowledge development, vocabulary enrichment, and engagement with grade-level syntax	■ Students read smaller sections of longer and complex grade-level text	Reading comprehension strategy instruction yielded positive improvements in students’ reading comprehension outcomes, with reported effects ranging from 0.30 to 0.97 (e.g., Daniel & Williams, 2019; Berkeley et al., 2010; Stevens et al., 2019)
		■ Pause and check for comprehension at the end of each section	
		■ Explicit instruction in strategy instruction can support students during reading	

The table summarizes the importance and recommendations for each component of reading instruction discussed in the article.

## PAUSE AND PONDER

- During reading, which reading-related tasks do my students find challenging?
- In designing a multicomponent reading intervention, which areas should I target to align with my students’ specific needs?
- How can I leverage my students’ strengths in certain reading tasks to foster the development of their other reading-related skills?

## TAKE ACTION!

### Implementing a Multicomponent Reading Intervention

1. Utilize the Explicit Instructional Framework: Start with modeling the activity followed by guided practice before expecting students to perform a task independently.
2. Adapt Lessons to Individual Needs: Tailor the instruction to match your students’ specific reading difficulties and diverse areas of reading needs. Implement multicomponent reading interventions during small-group sessions, addressing word reading, word meaning, reading fluency, vocabulary, and comprehension.
3. Incorporate Morphological Analysis: Teach word reading alongside word meaning, incorporating morphological analysis. Utilize images to enhance mastery of words among elementary school children with word reading difficulties.
4. Scaffold Reading Comprehension Activities: Divide grade-level passages into smaller sections for reflection and understanding. Teach reading strategies and keywords explicitly to facilitate main idea statement writing. Engage students in multiple readings of the same text to build background knowledge and ensure comprehension success.
5. Provide Corrective Feedback: Offer constructive feedback when students encounter challenges in writing main idea statements. Emphasize strengths and support students in identifying relevant keywords.
6. Monitor Progress: Regularly assess students’ reading fluency accuracy, word meaning growth, and reading comprehension. Record and graph students’ growth regularly.

By implementing these steps, educators can design and implement a successful multicomponent reading intervention that caters to the diverse needs of their students, promoting growth and proficiency in reading skills.

## MORE TO EXPLORE

**Table:** Resources for Developing Multicomponent Lessons

Resource type	Resource information
Example of multicomponent lessons	https://meadowscenter.org/resources/
Example of multicomponent lessons	https://www.readingresourcecentre.org/
In-depth review of each reading component along with example activities	https://ies.ed.gov/ncee/wwc/PracticeGuide/29
Reading-related activities for each component	https://fcrr.org/student-center-activities
Example lessons for each reading component	https://intensiveintervention.org
